# Impact evaluation of a maternal and child cash transfer intervention, integrated with nutrition, early childhood development, and agriculture messaging (MAZIKO-IE): a study protocol for a cluster-randomised controlled trial

**DOI:** 10.1186/s13063-023-07782-3

**Published:** 2024-01-13

**Authors:** The Maziko Trial Team, Aulo Gelli, Jan Duchoslav, Melissa Gladstone, Daniel Gilligan, Mangani Katundu, Ken Maleta, Agnes Quisumbing, Lilia Bliznashka, Marilyn Ahun

**Affiliations:** 1 International Food Policy Research Institute (IFPRI), 1201 Eye Street NW, Washington, DC USA; 2 University of Malawi, Zomba, Malawi; 3 University of Liverpool, Liverpool, UK; 4 Save the Children, Lilongwe, Malawi; 5 GiveDirectly, Lilongwe, Malawi

**Keywords:** Early child development, Nutrition, Cash transfers, Community-based child centres, Impact evaluation

## Abstract

**Background:**

Children in Malawi face high rates of malnutrition and are at risk of not reaching their developmental potential. Community-based childcare centres (CBCCs) can be cost-effective platforms for scaling-up early childhood development (ECD) and nutrition social behaviour change (SBC) interventions. However, evidence also suggests potential synergies from coupling nutrition SBC with cash transfers (CT), given that rural households in Malawi face high levels of poverty and recurring extreme lean season food-security shocks. The Maziko trial is aimed at evaluating the effectiveness and cost-effectiveness of using CBCCs and parenting care groups as platforms to improve maternal diets and child nutrition and development by providing nutrition-sensitive SBC and CT intervention packages in communities already receiving a standard of care Government SBC program.

**Methods:**

We designed a 3-year cluster-randomised controlled trial in two districts of Malawi, including 156 communities randomised to one of four treatment arms: (1) standard of care (SoC) arm: receiving the standard Government SBC program; (2) SBC arm: receiving the SoC intervention with additional nutrition-sensitive SBC activities to improve nutritious food production, diets, and care practices for young children; (3) low CT arm: SoC plus SBC plus a maternal and child cash transfer ~ 17 USD per month; and (4) high CT arm: SoC plus SBC plus a maternal and child CT ~ 43 USD per month. The trial will enrol pregnant women and children < 2 years of age. The primary outcomes are maternal diet assessed using the mean probability of adequacy and child development assessed using the Malawi Developmental Assessment Tool. Intermediate outcomes along the programme impact pathways will also be measured, including maternal mental health, maternal empowerment, child feeding practices, and child nutritional status.

**Discussion:**

This is the first study to examine the impact and synergies of combining ECD SBC with nutrition-sensitive SBC and CTs on maternal and child outcomes during the first 1000 days. The findings from this evaluation will inform national ECD and nutrition programmes.

**Trial registration:**

ISRCTN ISRCTN53055824. Registered on 7 March 2022.

**Supplementary Information:**

The online version contains supplementary material available at 10.1186/s13063-023-07782-3.

## Background

Over 250 million children are at risk of not reaching their developmental potential because of poverty, malnutrition, and other related adversities [1]. Estimates of the global burden of malnutrition indicate that undernutrition causes over 3 million child deaths per year [2] and that 155 million children aged < 5 years are stunted [3]. Micronutrient deficiencies contribute to both maternal and child mortality risks and affect children’s physical and mental development [4]. Malawi has one of the highest rates of chronic malnutrition in the world, with 37% of children aged 6–59 months being moderately or severely stunted [5]. Thirty-three percent of women and 63% of children < 5 years of age in Malawi are anaemic [5].

Early childhood development (ECD) involves interlinked physical, cognitive, and socio-emotional processes [6, 7]. Interventions designed to improve ECD have been shown to improve children’s cognitive, motor, and socio-emotional development [1, 8] and are considered among the most cost-effective investments in human capital [9]. Despite evidence on the effectiveness of ECD programmes, coverage and quality of implementation in low- and middle-income countries are low [1, 6]. In practice, ECD programmes are implemented through health, nutrition, education, and social protection sectors [10] and are most effective when targeting younger and more disadvantaged children and when also providing integrated health and nutrition services [6, 11]. Effective approaches also involve improving the quality of the learning environment and providing services to children directly. Training parents and involving them in practice and skill-building sessions are more effective than providing information alone [1].

Systematic reviews of the effects of cash transfers (CT) on maternal and child nutrition outcomes generally find consistent positive effects on food and nutrition intake and less consistent evidence suggesting small or null effects on micronutrient status and anthropometric outcomes, largely due to heterogenous programme designs [12, 13]. Evidence from CT programmes in Malawi includes an evaluation of the Social Cash Transfer Programme (SCTP), an unconditional CT programme targeted to ultra-poor, labour-constrained households, reaching all rural districts, with transfers ranging from 3 to 7 USD/month (2600–5600 MWK) depending on household size.^1^ A randomised controlled trial (RCT) of the SCTP found positive effects on household food consumption including increases in meal frequency, caloric availability, and reduction in the hunger gap but no impact on child anthropometry [14]. However, the SCTP is not been explicitly designed to address the multiple drivers of malnutrition and transfer levels remain low relative to the cost of living and are not indexed to inflation.

Evidence also suggests that well-implemented, nutrition-sensitive agricultural interventions have the potential to make important contributions to improving maternal and child nutrition outcomes [15, 16]. Findings from rigorous studies point to generally consistent impacts on dietary outcomes but less consistent impacts on children’s nutritional status, with scant evidence for other population groups. In Malawi, the NEEP-IE trial, an IFPRI/Save the Children RCT, compared the Government standard of care (SoC) behaviour change communication (BCC) package (including BCC on ECD, nutrition, and health) to SoC plus a more intensive nutrition-sensitive agriculture BCC package. The SoC package was equivalent to the treatment that was found to be most effective in a World Bank Protecting Early Childhood Development (PECD) four-arm trial [17]. The NEEP-IE trial showed that the nutrition-sensitive agricultural intervention implemented through community-based childcare centres (CBCCs) and parenting/care groups had large, positive effects on diets in pre-schoolers 3–6 years old and younger siblings 6–24 months old and on linear growth and stunting prevalence in younger siblings 6–24 months old [18]. A survey conducted 1 year post-trial showed that some benefits persisted (from improved nutrition to improved child development) whilst most other effects faded out. An economic evaluation found that the NEEP-IE programme had benefit–cost ratios from 3.6 to 24.7 [19]. Partially driven by the NEEP-IE trial findings, the Government of Malawi, through a grant from the World Bank, is in the process of scaling up components of the NEEP-IE package through the Investing in Early Years Program (IEYP) across 13 districts in the country [20].

The evidence from Malawi thus suggests that linking CBCCs and care groups can be a cost-effective platform for scaling up nutrition interventions. However, the literature also suggests potential synergies from coupling nutrition-sensitive BCC packages and CT [21]. Literature from Malawi also highlights that, in addition to high poverty levels, households also face recurring extreme lean season shocks when financial resources are typically most constrained. Theory would suggest that relaxing household budget constraints through unconditional CT may reduce vulnerability to price shocks, making the BCC package more effective. The scale-up of the IEYP in Malawi provides an ideal opportunity to support the roll-out and test the cost-effectiveness of additional, alternative approaches to improve infant and maternal nutrition. The Maziko study is a cluster-RCT (cRCT) designed to evaluate the impact and cost-effectiveness of an integrated SBC ECD package coupled with nutrition-sensitive BCC and CT on the diets, nutrition, and development of young children and their mothers in rural Malawi. This article describes the rationale, design, and methods for the Maziko trial.

## Methods

### Study objectives

The Maziko trial is aimed at evaluating the effectiveness and cost-effectiveness of using the integrated CBCC and parenting care-group platform to improve maternal and child nutrition and development by providing variations of nutrition-sensitive intervention packages in communities receiving standard Government support. The trial addresses one primary objective and several secondary objectives (Table 1).

**Table 1:** Objectives of the Maziko trial

Primary objective
To determine the independent effect of nutrition social behaviour change (SBC) and combined effects of nutrition SBC integrated with one of two levels of cash transfers on the adequacy of nutrient intake in women 15–49 years of age at baseline who are pregnant or mothers of children 0–2 years of age at baseline, and on child development (motor, language, and cognition) in children (in utero to 2 years of age at baseline), over a 3-year implementation period
Secondary objectives
1. To examine the effects of the randomised interventions on the Global Diet Quality Score in women 15–49 years of age at baseline who are pregnant or mothers of children 0–2 years of age 2. To examine the effects of the randomised interventions on the adequacy of nutrient intake in children in utero to 2 years of age at baseline 3. To examine the effects of the randomised interventions on height, height-for-age Z-score (HAZ), stunting (HAZ < − 2), weight, weigh-for-height Z-score (WHZ), wasting (WHZ < − 2), mid-upper arm circumference (MUAC), and head circumference in children in utero to 2 years of age at baseline 4. To examine the differential effects of the randomised interventions in the following pre-specified subgroups: a. Female and male children b. Intervention exposure timing/duration (pre-natal/post-natal) c. Subgroups formed by categorising household wealth, food security status, and maternal empowerment 5. To examine effects along the programme impact pathways (PIPs) linking programme implementation of each randomised intervention to nutrient adequacy of intake in women 15–49 years of age at baseline who are pregnant or mothers of children 0–2 years of age and children in utero to 2 years of age at baseline, and other outcomes by assessing: a. Fidelity of intervention implementation for each of the randomised interventions, defined as conformance with implementation specifications b. Quality of community-based childcare centres (CBCC) and care group training and supervision c. CBCC and care group volunteer capacity and service provision as related to the “All Children Surviving and Thriving” framework as assessed by questionnaire and observations d. Attained maternal knowledge and practices as related to the “All Children Surviving and Thriving” framework as assessed by questionnaire and observations 6. To examine the differential effects of the randomised interventions on women’s empowerment and maternal depressive symptoms, and how changes in these indicators mediate the effects on child-level nutrition and developmental outcomes 7. To examine the differential effects of the randomised interventions on household expenditure levels, shares of expenditures, poverty, and food-insecurity levels 8. To examine the differential effects of the randomised interventions on household food consumption elasticities for calories and micronutrients, what factors affect the consumption elasticities, and how changes in elasticities mediate the effects on child-level outcomes 9. To examine the differential effects of the randomised interventions on pregnant women and mothers of young infants’ willingness to pay (WTP) for nutrient-dense complementary foods, what factors affect the WTP for nutrient-dense complementary foods, and how changes in WTP mediate the effects on child-level outcomes 10. To assess costs and incremental cost-effectiveness of the randomised interventions compared to the standard of care 11. To examine the effects of the randomised interventions on haemoglobin concentration and anaemia prevalence in women 15–49 years of age at baseline who are pregnant or mothers of children 0–2 years of age and children in utero to 2 years of age at baseline (conditional on additional funding for data collection)

### Development of the interventions

The design of the interventions was guided by the “All Children Surviving and Thriving” systems framework, incorporating the Nurturing Care model and the UNICEF framework for the causes of malnutrition [7]. This framework includes five components that are necessary for children to thrive, including nutrition, health, opportunities for early learning, responsive caregiving, and security and safety. These proximal components are supported by the enabling environment extending from households to communities, programmes, and policies, which are influenced by distal factors including political, socio-economic, and climate change processes [22]. A key principle underpinning this system’s view of child development is that children’s well-being is responsive to modifiable factors including interventions and changes in the environment which can alter their developmental trajectories [1].

#### Standard of care

The national Nutrition Policy and Strategic Plan, led by the Department of Nutrition and HIV/AIDs, Ministry of Health, and Ministry of Gender, Children, Disability and Social Welfare of Malawi focuses on care groups, run by mother leaders and volunteers to reach pregnant and breastfeeding women and children < 5 years of age with key nutrition services, including home visits, cooking demonstrations, and promotion of essential services. The standard of care (SoC) will ensure the establishment of care groups and training of mother leaders and volunteers, through a cascade training, using the government-approved modules and materials. There are five modules in total: (1) breastfeeding; (2) complementary feeding; (3) hygiene; (4) maternal health and nutrition; and (5) ECD. These materials were recently updated to integrate ECD concepts across all materials. The cascade training was rolled out from national to district to teaching authority (TA) to community level targeting health and nutrition actors, including District Nutrition Coordinating Committees, Area Nutrition Committees, Village Nutrition Committees, Health Surveillance Assistants, and Care Group Promoters and Cluster leads (mother volunteers).

#### Nutrition-sensitive SBC interventions

To support uptake of recommended caregiving behaviours, a set of nutrition-sensitive SBC interventions will also be implemented. These included:

1. Food Security and Livelihood intervention

All households with pregnant and breastfeeding women and children < 5 years of age will be supported with training, seeds, and access to Village, Savings and Loans to help them grow more diverse and climate-resilient food and increase access to nutritious foods all year round. The CBCCs, where present and functional, will provide a platform for training, demonstration, and practice of new agricultural products and techniques, including food processing and utilisation techniques to reduce post-harvest loss, maximise nutrient content, and extend shelf life. Crop varieties that are fast-maturing and high-yielding will be prioritised, as well as those that require less water and can be grown in the dry season.

2) Gender transformative intervention

The REAL Fathers approach will be rolled out to the same households to improve the relationship between men and women and their children. REAL Fathers mentors are assigned four to five fathers and their wives to mentor through group meetings and home visits. Approximately seven topics are covered, including fathers’ vision of their family, their daughters and sons, the importance of good childcare and development, overcoming obstacles, family dreams, loving your family, and parenting [23]. The REAL Fathers approach will attempt to address issues identified in the Maziko project formative research, specifically non-engagement of fathers in childcare and maternal stress and exhaustion.

3) Adolescent intervention

Teenage pregnancy is very common in Malawi and one of the drivers of malnutrition. Adolescent girls and boys (with and without children) will be reached through youth clubs, using a peer-to-peer approach to change social norms around early pregnancy and encourage girls and boys to stay in school longer, delaying marriage and pregnancy. Girls with children will be supported to access existing services, including childcare through CBCCs and care groups. This approach will draw on existing approaches used in Malawi, such as youth empowerment and puberty books.

4) Reinforced ECD intervention

Additional training and support will be provided to care group promoters and cluster leads to reinforce ECD concepts, specifically responsive care, early stimulation, learning opportunities, maternal care, and care for children with feeding difficulties. This will include reinforcing the links between CBCC caregivers (teachers) and care group promoters. The Responsive Care and Early Learning Addendum materials will be adapted for Malawi and used as a “supplement” or addendum materials to the existing materials [24].

#### Cash transfers

The maternal and child cash transfer (CT) intervention contributes to Malawi’s National Social Support Policy of 2012 and the second Malawi National Social Support Programme (NSSP 2017–2022), by expanding CT beyond a poverty reduction-only goal to target pregnant women and mothers of young children. Enrolled women will receive one of two levels of monthly CT for the 3-year duration of the study. The CT will be provided through a mobile phone linked to a bank account registered with each trial participant. Upon registration, participants will be provided with a free mobile phone. The two CT values will be at ~ 17 USD per woman per month and ~ 43 USD per woman per month. The CT values were set based on an analysis of household consumption, poverty, and cost of diet in Malawi. Mean per capita consumption in rural Malawi was estimated at MWK 185,418 (~ 250 USD) [median MWK 151,157 (~ 200 USD)], per person per year, and the national poverty line in Malawi was set at 455 MWK (~ 0.6 USD) per person per day in 2020.^2^ Assuming an average household size of 4.4 members in Malawi, the proposed CT values are equivalent to 0.22 and 0.38 of the estimated monthly household expenditure. Both CT values are higher than the 0.2 rule of thumb threshold from cross-country analysis of the effectiveness of CT interventions [25]. A recent cost of diet analysis in Malawi found a lower bound cost of 1.75 USD (2011 US$ PPP) and upper bound cost of 2.26 USD per person per day [26]. This analysis also found that adequate diets were unaffordable to a minimum of 44% of the population in rural Malawi [26]. Thus, the smaller CT amount of ~ 17 USD per month will help fill the affordability gap for the mother and child’s nutritional needs, whereas the larger CT amount of 43 USD per month will help fill 50% of the affordability gap for the household nutritional needs. Following the completion of the randomised trial, the communities in the standard of care and SBC arms will also receive a one-off CT.

### Programme impact pathways

**Fig. 1:**
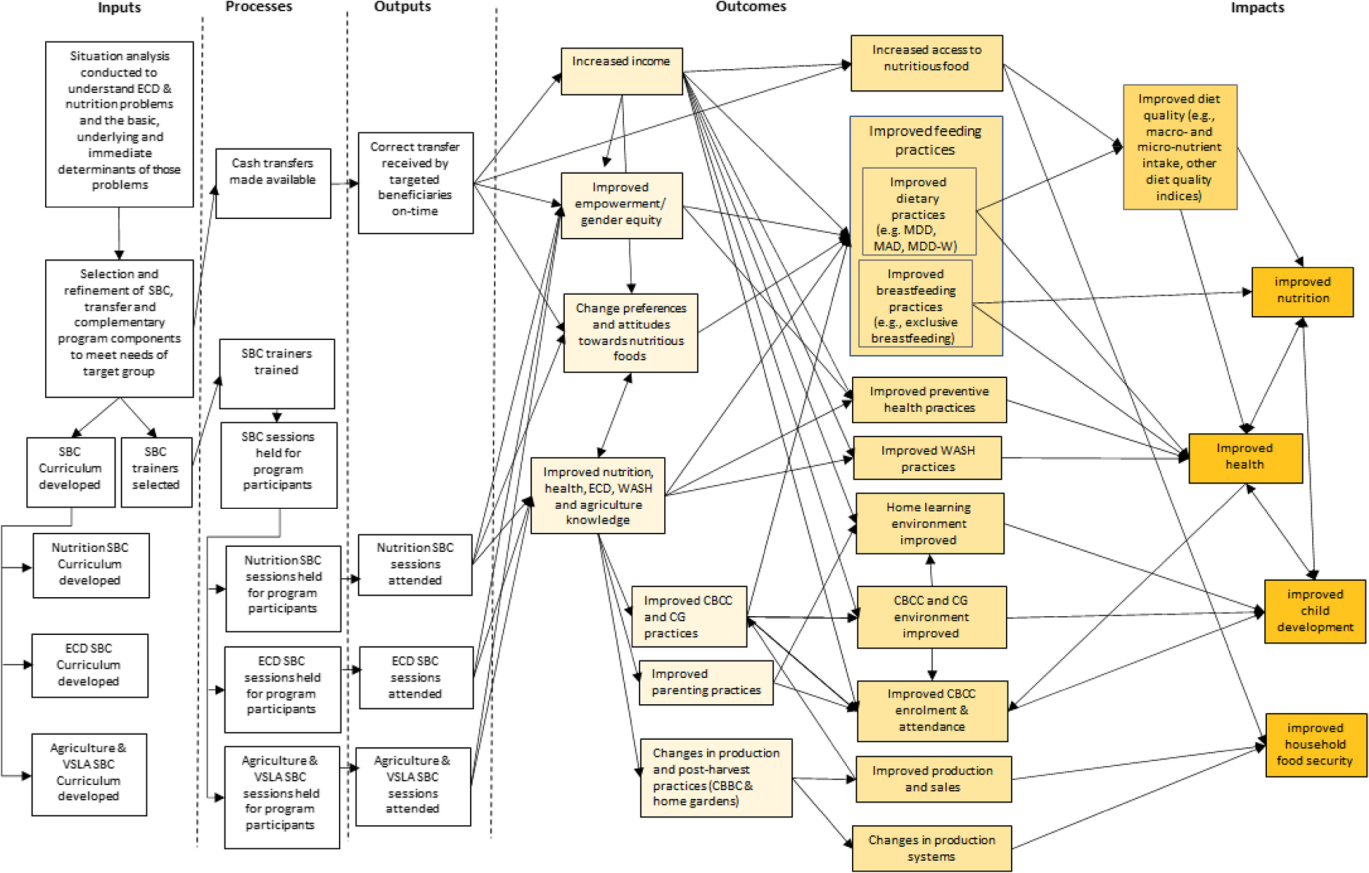
Stylised programme impact pathways for the MAZIKO intervention components

In this study, the care groups and CBCCs will be used as platforms to deliver an integrated ECD, agriculture, and nutrition SBC intervention targeting households with young children in the targeted communities. In addition, the parenting/care groups will provide a platform for CTs targeting pregnant women or mothers of children < 2 years old. The SoC intervention involves the establishment of Care Groups and a core set of training at the CBCC and parent level to improve breastfeeding and complementary feeding practices, hygiene, ECD, and maternal health and nutrition. The additional nutrition-sensitive SBC interventions can affect children’s development through two additional pathways (Fig. 1): (1) improved maternal and childcare practices (use of essential services, maternal nutrition, exclusive breastfeeding, age-appropriate complementary feeding, dietary diversity, hygiene, and child stimulation and bonding) through relevant, timely, and accurate advice and support for caregivers, adolescents, family members, their peers, and the wider community, and (2) improved household financial and physical access to nutritious foods all year round through improved farming practices, increased production, and improved crop production mix. The additional interventions focus on diets and nutrition could reinforce the focus on care and stimulation of young children, improving synergies across the two sectors, whilst increasing the salience of the core training and its relevance within households and the community. The CT intervention is expected to directly increase women’s income and empowerment and to reduce women’s depressive symptoms. The CT intervention is also expected to support changes in preferences of and attitudes towards nutritious foods. It could increase physical, emotional, and financial resources to channel towards health, nutrition, and development. Lastly, the CT intervention is expected to improve meals at home and in the CBCCs. By increasing the quality and regularity of meals in the CBCC, the intervention could influence young children’s CBCC participation as they grow older, potentially enhancing both their development and nutritional status. Implementing the interventions will not require alteration to usual care pathways (including the use of any medication) and these will continue for all trial participants.

### Study sites

#### Study site selection and description

The study site includes the two contiguous districts of Balaka in the Southern region and Ntcheu in the Central region of Malawi. Districts were selected based on the following criteria: (1) having higher stunting rates than the national average, (2) identified service delivery gaps, (3) the presence of implementing partners, and (4) priority districts for the Government.

#### Cluster formation

The study area includes 156 clusters, defined as communities including the catchment area of CBCCs targeted by the interventions. Location and basic infrastructure information was collected from the IEYP CBCC mapping exercise obtained from the Ministry of Gender, Children, Disability and Social Welfare for Ntcheu. For Balaka, a CBCC mapping was conducted by Save the Children in December 2021, including GPS location and data on infrastructure and recent functioning.

#### Community engagement

The targeted communities include CBCCs that have been part of the national ECD policy and programme. Save the Children has been working in the ECD domain in Malawi including support to CBCCs. The project kick-off meetings with Government of Malawi stakeholders at central and district levels from the relevant ministries and implementing partners were undertaken in December 2021. Community-level engagement included kick-off sensitisation meetings at CBCC level prior to randomisation.

#### Study population

The RCT study population includes pregnant women or mothers of children < 2 years of age at baseline living in CBCC catchment area. All women with self-reported pregnancy or mothers/caregivers of children aged < 2 years will be eligible to participate in the programme at the cluster level. Study inclusion criteria are as follows: (1) all women aged 15 to 49 years who are pregnant living in the catchment area of the CBCC whose pregnancy is confirmed by a urine pregnancy test, and (2) mothers (aged 15 to 49 years) of children aged < 2 years of age living in the catchment area of the CBCC. Women residing in the study area who are pregnant during the enrolment period but do not consent to participate in the trial will be excluded. Children with major non-fatal disabilities will not be excluded from study procedures but will be excluded from the final analysis sample if the disability is likely to directly affect growth and development. There will be no special criteria for discontinuing or modifying allocated interventions.

### Sample size and power

The primary outcomes of the trial include women’s diet assessed using the mean probability of adequacy (MPA) of micronutrient intake and child development (motor, language, and cognitive) assessed using the Malawi Developmental Assessment Tool (MDAT). Power calculations based on available clusters in targeted districts and resource availability suggested ~ 40 clusters per intervention arm and 20 households (mother–child pairs) per cluster. Calculations based on MPA and a cRCT design (inter-cluster correlation coefficient (ICC) = 0.05) suggested a Minimum Detectable Effect Size (MDES) of 0.31 SDs. For reference, the effect of the nutrition SBC intervention found during NEEP-IE trial on MPA of children 3–6 years old was 0.34 SDs [27]. Similar calculations for MDAT *Z*-score suggested an MDES of 0.26 SDs (ICC = 0.02). The effect of the SBC intervention on children 6–24 months of age found after a 2-year period (with only 1 year with intervention) during the NEEP-IE trial was 0.23 SDs.

### Randomisation and masking

Clusters were randomly assigned to one of four intervention arms using a restricted randomisation procedure [28]. Restricted randomisation involves modelling selection using a set of village-level variables to ensure balance across the intervention arms. The variables in the restricted randomisation were selected based on their potential influence on the main study outcomes and their potential influence on participation in the interventions [28]. The village-level variables included in the model were population size, total per capita household expenditure, household dwelling state, household drinking water source, women’s empowerment, maternal caloric intake, maternal micronutrient intake, maternal age, pregnancy status, maternal weight, maternal education, maternal marital status, child age, child sex, child weight, child height, and child and total MDAT score. Household-level data were used to generate village-level aggregates for all variables except for population size which was available at the village level. We developed a programme using Stata 17 [29] to randomly allocate clusters to four different arms stratifying by district. The randomisation was stratified by district to allow for the same approximate number of clusters within each of the intervention arms in each of the two districts. The algorithm then regressed selection into the intervention arm on the village-level variables. The algorithm tested 5000 random allocations and then selected the permutation that minimised the *R*^2^ for the predicted selection. Description of the intervention arms is provided in Table 2.

**Table 2:** Description of the four intervention arms

Intervention arm	Care group component	SBC component	Cash transfer component	Number of clusters
Standard of care (SoC)	Yes	No	No	39
Nutrition-sensitive social and behaviour change (SBC)	Yes	Nutrition-sensitive SBC on agriculture, gender, adolescence, and extra ECD	No	39
SBC + low cash	Yes	Nutrition-sensitive SBC on agriculture, gender, adolescence, and extra ECD	~ $17 per month	39
SBC + high cash	Yes	Nutrition-sensitive SBC on agriculture, gender, adolescence, and extra ECD	~ $43 per month	39

Given the nature of the intervention, participating women, children, and households were not blinded to the intervention assignment. Neither were intervention implementers. The field team, including the study coordinator, supervisor, and enumerators, will be blinded to intervention allocation at the midline and endline surveys. Data analyses conducing impact analyses using midline and/or endline data will be blinded to intervention assignment. A non-study statistician will unblind the results once finalised.

### Recruitment of subjects

Prior to the baseline survey, a household census was conducted in the CBCC catchment areas which collected information on household demographics and self-reported pregnancy status. Data from the census were used to construct a listing of households for the survey sample with women aged 15–49 years who self-report as pregnant and/or were mothers of children 0–2 years of age. An index dyad (a woman who is pregnant or a mother with a child aged 0–2 years and her child 0–2 years old) were randomly selected for inclusion in the baseline survey sample, stratifying the random selection by pregnancy status. During the baseline interview, information was collected on the last menstrual period date. Women with confirmed pregnancies not enrolled in ante-natal services were referred to village health agents and antenatal services. The baseline survey sample listing included potential replacements to ensure adequate sample sizes were obtained.

### Retention and follow-up

All questionnaires will be administered to enrolled participants at follow-up surveys (upon obtaining informed consent) even if they discontinue participation in the interventions or deviate from intervention protocols. Reasons for discontinuing participation will also be collected.

### Outcome measurement

The study involves survey rounds at baseline, midline, and a 3-year follow-up, including questionnaires at village/CBCC, household, maternal, and child levels (Table 3). The surveys will be conducted by enumerators trained over a 3-week period on research ethics and good clinical practices, questionnaire comprehension, interviewing techniques, data entry on tablets using CAPI software, multi-pass 24-h dietary recall, child development using MDAT, maternal and child anthropometry, and study standard operating procedures. Training and standardisation exercises will be conducted to assess reliability, intra- and inter-observer accuracy as compared to gold-standard child development and anthropometry specialists. Enumerators will be grouped into specialised teams (including household interviews and dietary assessment, child development and anthropometry). Supervisors will conduct interview spot-checks during the survey, troubleshoot any issues that may arise during data collection, and debrief with each team at the end of each day.

**Table 3:** Measurement schedule for primary and secondary outcomes

	**Data collection round**		
**Data collected**	**Baseline**	**Mid-line (1.5 years)**	**Endline (3 years)**
*CBCC/village level*			
Village infrastructure and development projects	I		I
CBCC infrastructure and staff	I		I
Parenting/care groups	I		I
Market prices	I	I	I
*Household level*			
Demographics, socioeconomic status	I		I
Expenditures	I	I	I
Agriculture production	I	I	I
*Maternal level*			
Programme exposure	I	I	I
Women's empowerment (Pro-WEAI)		IDI/FGDs	
Role in household decision-making around production and income	I	I	I
Access to productive capital	I	I	I
Access to financial services	I		I
Time allocation	I		I
Group membership	I		I
Physical mobility	I		I
Intrahousehold relationships	I		I
Autonomy in decision-making	I		I
New general self-efficacy scale	I		I
Life satisfaction			
Attitudes about domestic violence	I		I
Maternal depressive symptoms (SRQ)	I	I	I
Height and weight	M	I	M
Haemoglobin (conditional on additional funding)			B
24-h dietary recall	I	I	I
IYCF knowledge and practices	I	I	I
Willingness to pay for specialised foods	I	I	I
*Paternal level*			
Women’s empowerment (Pro-WEAI)	I	IDI/FGDs	I
*Child level*			
Birth information (date, time, mode, place, complications, care, weight)	R	R	
Immunisation and vitamin A	I	I	I
7-day morbidity symptoms, health record (clinic visits, hospitalisations)	I	I	I
Height and weight	M	I	M
Child development (MDAT)	I/O		I/O
Family care indicators	I	I	I
24-h dietary recall			I

*Abbreviations: B*, blood; *CBCC*, community-based childcare centre; *FGD*, focus group discussion; *I*, interview; *IDI*, in-depth interview; *IYCF*, infant and young child feeding; *M*, measurement; *MDAT*, Malawi Development Assessment Tool; *O*, observation; *SRQ*, self-reported questionnaire; *T*, test; *WEAI*, women empowerment in agriculture index

#### Dietary assessment

Dietary assessment will be undertaken in index women and children using the multi-pass, 24-h recall method [30]. This includes the following: (First pass), respondents recall the list of all foods, drinks, and snacks consumed during the previous 24 h; (Second pass) precise descriptions of all foods consumed are obtained, including recipes and details for all mixed dishes; (Third pass), respondents estimate portion sizes using pre-defined methods for each of the foods consumed, including weighing a replica, volume measurements, using clay, wooden or plastic models, calibrated household measures, or by collecting costs of actual purchases. Once the recall is finished for index mothers, enumerators will repeat the process with caregivers to recall the food consumption of the index child. Finally (Fourth pass), once the description of foods is complete for both the woman and the index child, the enumerator verifies with mothers all foods and quantities consumed by both themselves and their child the previous day. A second recall will be conducted in approximately 10% of the sample on a non-consecutive day by a different enumerator. Food intakes will be converted to nutrients and the prevalence of adequacy of nutrient intake will be estimated using the NCI method to estimate the population usual intake distributions using the two recall days to adjust for within-person variation [31].

#### Child development assessment

The Malawi Developmental Assessment Tool (MDAT) will be used to assess the child motor, language, and cognitive development [32]. The short Washington Group/UNICEF child functioning questionnaire will be administered to assess functional difficulties in hearing, vision, comprehension, and mobility [33]. At endline, additional cognitive assessment tools will also be considered. Domain-specific and aggregate child development scores will be calculated using standard procedures [32]. The MDAT tool includes norms for Malawian children that are used to convert the raw MDAT scores to *Z*-scores. An additional cognitive test (e.g. standard progressive matrices, digit span, blocks) will be also considered at endline to mitigate potential tail effects from ageing-out of MDAT.

#### Anthropometry assessment

Child weight will be measured to the nearest 100 g using an electronic scale. Recumbent length of children < 2 years of age and standing height of children > 2 years of age will be measured to the nearest 0.1 cm using length boards or portable fixed base stadiometers. All measurements will be undertaken in duplicate by an anthropometrist and an assistant. Maternal weight and height will be measured using electronic scales and stadiometers, respectively.

#### Birth information in sub-sample of pregnant women

The sample of pregnant women will be followed-up post-baseline by field monitors accompanied by village health agents to collect birth information including, childbirth date, delivery mode, complications, birth weight, birth length, and head circumference from institutional registers and the mother’s antenatal cards. For women who delivered at home, visits will be conducted to collect birth information and newborn anthropometric measurements if the research team learns of the delivery within 7 days. Birth dates will be combined with the last menstrual period dates to calculate gestational age at birth and to categorise preterm births.

#### Process monitoring and evaluation

A theory-driven process evaluation combining qualitative and quantitative data will be undertaken at midline approximately 18 months after the baseline to provide evidence of changes along the programme impact pathways. We will work closely with the programme implementers to identify indicators for the key processes and programme impact pathways for the intervention packages. Monthly and quarterly monitoring data from programme implementers will be examined to examine adherence and provide feedback to communities to encourage participation. In addition, the qualitative research component will focus on understanding the perceived drivers of change along the programme impact pathways and the barriers related to social and cultural norms, especially for women. Selected outcome-level data likely to be affected by large seasonal changes, including dietary intake and expenditures, will also be measured on a random sub-sample of the study population.

#### Formative research

Qualitative, in-depth research will be undertaken to understand existing caregiving behaviours, identify individual, household, service, social and environmental determinants of caregiving behaviours (barriers and motivations), and inform the design of the SBC intervention. This will include multi-day immersion by Save the Children trained staff in communities, living with families and informal conversations, observation, and participation in daily household activities, followed by co-creation workshops with communities. Qualitative data will be translated, transcribed, and thematically coded using a set of predetermined codes based on the research objectives and the PIP framework. Codes will then be organised according to common emergent themes and framework matrices will be used for thematic and explanatory analysis.

### Observational studies of other outcomes

A number of observational studies on nutrition, disability, poverty, and developmental dynamics will be conducted in the framework of the cRCT, including a validation study of the Global Diet Quality Score as a proxy for nutrient intake in women, cost of diet, and cohort studies that will include the randomised interventions as covariates in the analysis.

### Economic evaluation

**Fig. 2:**
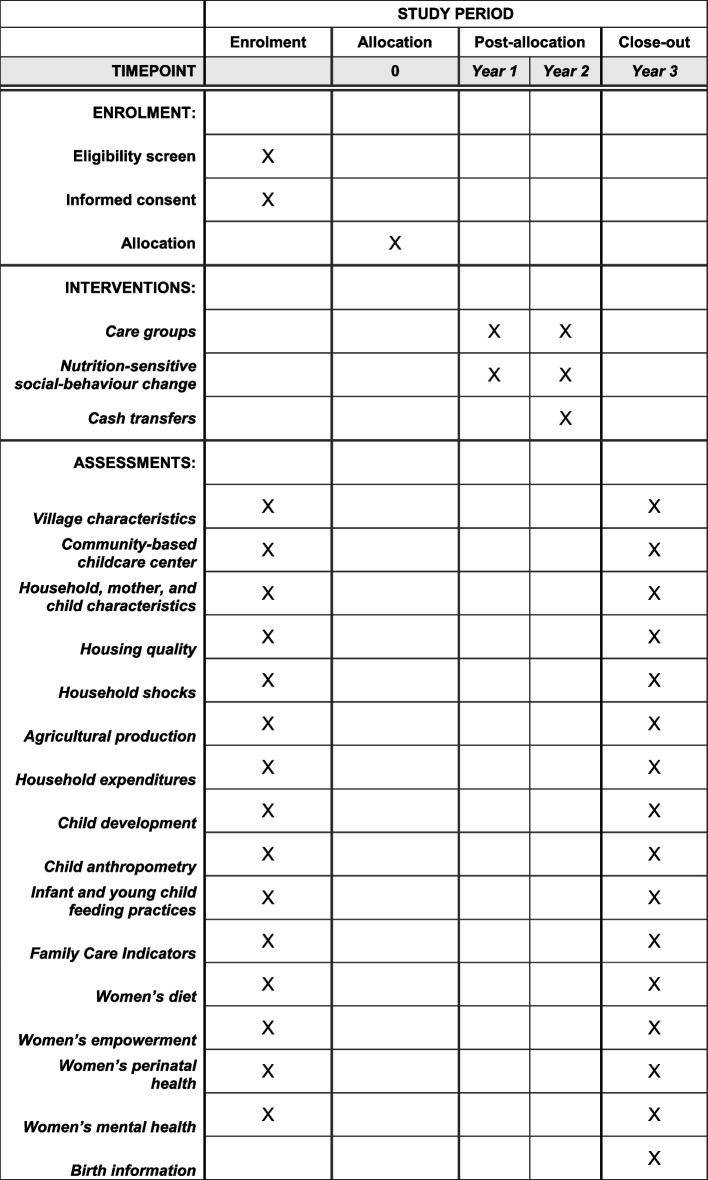
Schedule of enrollment, interventions, and assessments (SPIRIT Figure)

We will estimate the financial and economic costs of the interventions and combine economic costs with measures of impact to estimate the incremental cost-effectiveness ratio (ICER) of the intervention compared to the status quo (the standard of care). The ICER is defined as the incremental cost per outcome gained. We will apply a standardised framework for understanding the costs and benefits of multisectoral nutrition strategies that build on best practices in conducting cost, cost-effectiveness, and benefit–cost analyses [34, 35]. For cost data collection, we will capture all costs incurred by implementing partners, for both inputs (personnel, capital equipment, supplies, overhead) and activities (start-up and disaggregated recurrent activities). We will use a mixed methods approach that combines using financial expense reports obtained by project records, and micro-costing for all resources used that are not captured in the financial analysis. We will capture information on time use by project staff using interview and focus group discussion. Similarly, we will collect time use data and out-of-pocket expenses for volunteers and project participants. We will combine financial and economic costs to estimate the cost per participant using an Excel-based costing model. We will use standard cost-effectiveness methods to estimate incremental costs with net benefits. See Fig. 2 for schedule of enrolment and intervention assessment (SPIRIT Figure).

### Data flow and management

All data will be collected using tablets preloaded with questionnaires programmed using CAPI software (Survey CTO). Data collection will include creating anonymised versions of the datasets. The data files in Stata format containing identifying information from the baseline survey will be held only on the principal investigators’ (PIs) computer, for use in tracking and identifying baseline survey respondents during the follow-up surveys. The data will be processed, cleaned, and documented by research assistants working under close guidance of the Pis. The documentation will include questionnaires, data variable labels, value labels, and a codebook to assist in the analysis. Field notes from the fieldwork coordinator and team leaders describing the data collection process will also be stored. Within 12 months of the end of the project, the anonymised data will be made publicly available on the IFPRI website. Users of the data will be asked to provide information on how the data are expected to be used to help IFPRI track the use of the data for research purposes.

### Data monitoring

Adverse events (AEs), serious adverse events (SAEs), or harms from the interventions are not anticipated. There is no anticipated harm and compensation for trial participation. Minor AEs or harms may include community disagreements related to some community members receiving the interventions and others not receiving them. Data on such events will be collected at follow-up surveys. In addition, we will collect data on SAEs in pregnant women (e.g. miscarriage, stillbirth) and children (e.g. child death, severe morbidity) at follow-up surveys to assess any potential negative unintended consequences of the interventions. Interim analyses based on programme monitoring and midline evaluation, including potential unintended effects, will be presented to the trial steering group within 3 months of the midline survey completion. The trial steering group will sign off the study design, interim, and final analyses.

### Statistical analysis

The randomised design allows for the identification of causal impacts of the interventions using comparisons of mean outcomes between the randomised intervention arms at endline. The analysis will follow the intention-to-treat approach, using econometric analysis to control for differences at baseline across intervention arms for all variables of interest. Following Bruhn and McKenzie [36], impact will be assessed using a single difference analysis of covariance (ANCOVA) model with the following form:

$${Y}_{i1}={\beta }_{0}+{\beta }_{1}{T}_{i}+{{\beta }_{2}Y}_{i0}+{\varepsilon }_{i}$$

where ${Y}_{i0}$ is the outcome variable at baseline, ${Y}_{i1}$ is the outcome variable at endline, and ${T}_{i}$ is a dummy variable for treatment status. The ANCOVA estimator has been shown to provide more efficient estimates of programme impact than difference-in-difference models when auto-correlation of outcomes is low [36]. To account for the cRCT design and the level of clustering of the outcome under analysis, we will employ multi-level regression models [37]. The multi-level models will use both fixed effects with dummy variables for each intervention and random effects at the cluster level (unit of randomisation) to account for clustering and to estimate the standard error in an unbiased manner. Alternative fixed effect models with standard errors clustered at the cluster level will also be considered. Primary analyses will be unadjusted for baseline covariates. In addition, we will report adjusted estimates, controlling for baseline covariates. We will also adjust for the study district to account for any agro-ecological differences that may influence the food security and livelihood interventions. The purpose of the adjustment is to account for imbalances at baseline and to reduce variance. The intent-to-treat analysis strategy will include attempts to follow up all individuals in the study, the development of a main analysis that is valid under a stated plausible assumption on missing data, and sensitivity analysis to explore the effects of departures from the assumption underlying the main analysis. Data management, data cleaning, and statistical analyses will be conducted using Stata, SAS, and R. Results will be reported following the guidelines established in the CONSORT guidance for cRCTs [38].

## Discussion

ECD programmes are considered among the most cost-effective investments in human capital [9], resulting in improvements in children’s cognitive, motor, and socio-emotional development [1, 8]. Nutrition-sensitive agricultural programmes, when well-implemented, can improve maternal and child dietary outcomes and, in some cases, children’s nutritional status [15, 16]. Further, CT programmes can improve food and nutrition intake, but have more inconsistent effects on micronutrient status and anthropometry [12, 13]. Combining these sectors may lead to substantial gains in effectiveness, efficiency, and cost. Existing evidence highlights the potential for additive and synergistic effects when combining ECD, nutrition, agriculture, and CT interventions [11, 16, 39, 40]. However, there is a need to rigorously assess these potential synergies. This article describes the rationale, design, and methods for the Maziko trial, a cRCT designed to evaluate the impact and cost-effectiveness of an integrated SBC ECD package coupled with nutrition-sensitive SBC and CT on the diets, nutrition, and development of young children and their mothers in rural Malawi. To our knowledge, this is the first study to examine the synergistic effects of combining ECD, nutrition, agriculture, and CT interventions on women’s and children’s diet, nutrition, and development outcomes.

The Maziko study will provide evidence on the effectiveness and cost-effectiveness of integrating ECD, nutrition, agriculture, and CT interventions to support households with young children in rural Malawi. The trial will inform the scaling-up of these services in Malawi. Results are likely to be generalisable to similar rural settings in sub-Saharan Africa with high poverty levels, where households face recurring extreme lean season shocks and constrained financial resources for child diets, nutrition, and development.

## Trial status

Protocol version 4, 12/09/2023. The first participant was enrolled on 2 May 2022 and recruitment was completed on 11 July 2022. Data collection will be completed by June 2025.

## Protocol amendments

Protocol amendments are not planned. If any important protocol modifications are required (such as changes in eligibility criteria, outcome, or analyses), these changes will be reported to the institutional review boards providing ethical clearance for the study. The trial registration will be updated accordingly once approvals are received.

## Dissemination policy

Study findings will be disseminated through in-person workshops with stakeholders in the villages/clusters, districts, and regions where the study is to be conducted; in-person and virtual workshops with key local, national, and international stakeholders; and policy briefs, blog posts, social media posts, peer-reviewed publications, and presentations at scientific and programmatic meetings. All peer-reviewed publications will also be added to the trial registration website.

All authors of peer-reviewed publications must make a substantive contribution to the intellectual content according to the ICMJE authorship criteria. All peer-reviewed publications will contain CRediT (Contributor Roles Taxonomy) author statement or alternative author contribution statement based on journal requirements.

### Supplementary Information

**Additional file 1.**

## Data Availability

All members of the Maziko trial team will have access to the final de-identified datasets. Only the PI will retain identifying data on an encrypted password-protected computer for use in tracking and identifying baseline survey respondents during the follow-up surveys. All identifying information will be deleted within 12 months of completing the project. The anonymised data will be made publicly available on the IFPRI website within 12 months of the end of the project. Users of the data will be asked to provide information on how the data are expected to be used to help IFPRI track use of the data for research purposes. Any data required to support the protocol can be supplied on request. The statistical code to replicate findings from individual papers can be made available upon reasonable request to the corresponding author for the paper.

## Abbreviations

ANCOVA: Analysis of covariance
BCC: Behaviour change communication
CAPI: Computer-assisted personal interviewing
CBCCs: Community-based childcare centres
cRCT: Cluster-randomised controlled trial
CT: Cash transfers
ECD: Early childhood development
GoM: Government of Malawi
ICER: Incremental cost-effectiveness ratio
IEYP: Investing in Early Years Program
MDAT: Malawi Development Assessment Tool
MDES: Minimum detectable effect size
MPA: Mean probability of adequacy
NEEP-IE: Nutrition Embedded Evaluation Programme Impact Evaluation
PECD: Protecting Early Childhood Development
RCT: Randomised controlled trial
SBC: Social behaviour change
SCTP: Social Cash Transfer Programme
SoC: Standard of care

## Footnotes

1 The size of the SCTP transfers has increased over the years. The 2020 figures, referring to the basic amount which depends on household size, are as follows: 2600 MWK for one-person households; 3300 MWK for two-person households; 4400 MWK for three-person households, and 5600 MWK for four-plus-person households. Additionally, the programme includes a bonus for each child attending school, originally of the order of 500 MWK and 1000 MWK for primary and secondary school pupils, respectively. It is unclear from official documentation if these schooling incentives increased along with the standard transfer amounts. Recent analysis by UNICEF reports that the total monthly transfer is worth on average 7000 MWK (~ 10 USD in 2019) per household.

2 Values for consumption in the two districts where the trial will take place (Balaka and Ntcheu) are lower than those reported at national level, or approximately 0.92–0.98 times consumption at national level.
